# Keep Away from Danger: Dangerous Objects in Dynamic and Static Situations

**DOI:** 10.3389/fnhum.2013.00344

**Published:** 2013-07-02

**Authors:** Filomena Anelli, Roberto Nicoletti, Roberto Bolzani, Anna M. Borghi

**Affiliations:** ^1^Department of Philosophy and Communication, University of Bologna, Bologna, Italy; ^2^Department of Education Sciences, University of Bologna, Bologna, Italy; ^3^Department of Psychology, University of Bologna, Bologna, Italy; ^4^Institute of Cognitive Sciences and Technologies, National Research Council, Roma, Italy

**Keywords:** dangerous objects, affordances, space, dynamic and static presentation, motor system, conceptual development, dynamic affordance effect, escaping/avoidance effect

## Abstract

Behavioral and neuroscience studies have shown that objects observation evokes specific affordances (i.e., action possibilities) and motor responses. Recent findings provide evidence that even dangerous objects can modulate the motor system evoking aversive affordances. This sounds intriguing since so far the majority of behavioral, brain imaging, and transcranial magnetic stimulation studies with painful and dangerous stimuli strictly concerned the domain of pain, with the exception of evidence suggesting sensitivity to objects’ affordances when neutral objects are located in participants’ peripersonal space. This study investigates whether the observation of a neutral or dangerous object in a static or dynamic situation differently influences motor responses, and the time-course of the dangerous objects’ processing. In three experiments we manipulated: object dangerousness (neutral vs. dangerous); object category (artifact vs. natural); manual response typology (press vs. release a key); object presentation (Experiment 1: dynamic, Experiments 2 and 3: static); object movement direction (Experiment 1: away vs. toward the participant) or size (Experiments 2 and 3: big vs. normal vs. small). The task required participants to decide whether the object was an artifact or a natural object, by pressing or releasing one key. Results showed a facilitation for neutral over dangerous objects in the static situation, probably due to an affordance effect. Instead, in the dynamic condition responses were modulated by the object movement direction, with a dynamic affordance effect elicited by neutral objects and an escape-avoidance effect provoked by dangerous objects (neutral objects were processed faster when they moved toward-approached the participant, whereas dangerous objects were processed faster when they moved away from the participant). Moreover, static stimuli influenced the manual response typology. These data indicate the emergence of dynamic affordance and escaping-avoidance effects.

## Introduction

In our lives we constantly interact with different kinds of objects, characterized by different features, and we need to learn their properties. For example, so far literature investigated the importance of size (e.g., Tucker and Ellis, 2001), shape (e.g., Smith, 2005; Panis et al., 2008a,b), weight (e.g., Brouwer et al., 2006; Scorolli et al., 2009), and consistence (Anelli et al., 2010) for categorization. Among different properties of the objects, dangerousness can be considered of particular relevance for our survival. This implies that the study of the ability to discriminate between objects we can interact with and we can eventually use without any problem, and objects that can provoke pain represents an interesting and growing research field. We will call the first neutral objects, the second dangerous objects. Notice that, in keeping with the studies on dangerous objects we will briefly review, we will use a rather broad definition of object dangerousness. We define as dangerous those objects and entities that can provoke harm, independently of whether this harm is intentionally or accidentally provoked. Hence, we consider dangerous both a scorpio who approaches us and a cactus which can potentially hurt us when we approach it.

Since Gibson (1979) proposed a theory of affordances, defining them as properties in the environment that are relevant for an organism’s goals, the notion that objects are represented in terms of potential actions (i.e., affordances) has gained growing interest. To clarify with an example, a cup provides affordances, i.e., it “invites” us to act, for example to reach and grasp its handle. More recent theorization on affordances conceived them as “brain assemblies” that represent objects, that is as the result in the brain of the connection between visual and motor responses that have developed during the adaptation to the physical and social environment (Ellis and Tucker, 2000). Over the past decade, a growing number of cognitive and neuroimaging studies has focused on affordances, and computational models have been proposed (for a recent review, see Thill et al., 2013). Data from neurophysiological and neuroimaging studies, both on monkeys and humans, revealed that specific parieto-frontal circuits are responsible for the encoding of the observed features in terms of action potentialities. In monkeys, the so-called “canonical neurons,” that probably constitute the neural basis of affordances, were activated even when the monkey simply observed a graspable object and thus no overt response was required (e.g., Jeannerod et al., 1995; Murata et al., 1997; Raos et al., 2006; Umiltà et al., 2007).

As for humans, similar results have been obtained with brain activation studies (for a review, see Martin, 2007). For example, in a seminal PET study, Grafton et al. (1997) registered the automatic activation of the action observation network (i.e., the dorsal premotor cortex and the anterior intraparietal sulcus) during the mere observation of manipulable objects such as tools, even in the absence of overt motor response. Further fMRI studies demonstrated the activation of a fronto-parietal circuit (i.e., the left premotor cortex and the inferior parietal lobule) when graspable objects were observed (Chao and Martin, 2000) and during the execution of a specific hand grip posture, on the basis of the specific hand grip posture afforded by the object features (Grèzes et al., 2003).

In addition to these findings, several cognitive behavioral studies have demonstrated that overt reaching and grasping movements can be activated during objects observation (for reviews, see Borghi and Cimatti, 2010; Borghi et al., 2012).

One line of research particularly relevant to the issue addressed in our study concerns the relation between affordances and space. In a series of studies, Costantini and colleagues tried to clarify whether affordances differently emerged when objects, as for example bottles, were located within or outside the perceiver’s peripersonal space, namely in the space that encompassed the objects within reach (Rizzolatti et al., 1997). In a first behavioral experiment, Costantini et al. (2010) employed a spatial alignment effect paradigm, requiring participants to replicate a grasping movement as soon as a go-signal became visible (i.e., a mug’s handle, placed either within or outside the participants’ reaching space). The study revealed that participants responded to affordances only when the object was in the observer’s peripersonal space, and thus in her reachable space, and not when it was located in her extrapersonal space (see also Costantini et al., 2011a, for a replication of the same effects in a task in which not only images of objects but verbs were used as well; see Coello and Bonnotte, 2013, for an investigation of the link between the spatial content of determiners and the spatial representation of action possibilities). In a subsequent behavioral study, Costantini et al. (2011b) used the previous paradigm but introduced in half of the trials the presence of an avatar. They expanded previous results demonstrating the presence of an affordance effect even when the object was outside the observer’s reachable space, provided that it was located within another individual’s reaching space. For example, when a mug was located in the participant’s far space but it was close to the avatar, the affordance effect was present. These findings were also supported by transcranial magnetic stimulation (TMS) studies (Cardellicchio et al., 2011, 2012).

Another line of research deserves to be introduced, namely studies aimed at investigating responses induced by the observation of others’ pain. In a seminal study, Singer et al. (2004b) measured empathic brain activations *in vivo* with fMRI, by registering brain activity in the female partner of couples of participants. Painful stimulation was applied either to her own hand, thus measuring pain-related brain activation of the felt pain, or to her partner’s hand, thus measuring pain-related brain activation of the empathy for pain. The results revealed the activation of bilateral anterior insula and anterior cingulate cortex, i.e., of parts of a complex neural network (the so-called “pain matrix”): the pain matrix was activated both when subjects experienced pain themselves and when they saw a signal indicating that the partner had experienced pain. Activation in this network was also registered when subjects watched videos showing body parts in potentially painful situations (Jackson et al., 2006), painful facial expressions (Lamm et al., 2007), or hands being pricked by needles (Morrison et al., 2004, 2007a). Further studies suggested that the magnitude of these empathic brain responses can be modulated by different factors, such as the perceived fairness of the other (Singer et al., 2004a, 2006) and the intensity of the inflicted pain (Avenanti et al., 2006; for a review, see de Vignemont and Singer, 2006).

In addition, in a series of TMS studies Avenanti et al. (2005, 2006) explored passive responses during pain observation. By measuring motor-evoked potentials (MEPs), results demonstrated a specific corticospinal inhibition when observers watched someone else suffer a painful stimulation (i.e., watching a needle inserted deep into a model hand). Indeed, the significant MEPs amplitude decrease was specific for the observed body part (i.e., for the hand and not for the foot) and for the involved muscle, while it was not present when the needle was inserted into a tomato (not body part) or when the hand was given to a tactile stimulation (innocuous cotton bud). Thus, pain observation led to a specific corticospinal inhibition, similar to directly experienced painful stimulation (e.g., Le Pera et al., 2001; Farina et al., 2003). This finding suggested an activation of pain representations in the observer’s sensorimotor system due to motor resonance. This pointed out an important role of motor areas in the pain matrix, both during the first-person experience of pain and during empathy for others’ pain.

Not only neural, but also behavioral evidence demonstrated a specific influence of pain observation on overt motor responses. A study of Morrison et al. (2007b) showed that observing a video of a painful stimulation (i.e., a needle penetrating a hand) speeded withdrawal movements (key-releases) and slowed approach movements (key-presses); this difference was not present when participants observed a neutral stimulation (i.e., a cotton bud touching a hand) or when both the painful and neutral stimulation concerned a non-biological (i.e., a sponge) rather than a biological stimulus (i.e., a hand).

On the whole, these findings reveal the emergence of resonance mechanisms when pain was passively induced by an object and participants could observe the direct interaction between a hand and a needle (see Haggard et al., 2013, for a review on the link between brain mechanisms of pain and its perceptual quality with the spatial structure of the body).

Thus, so far several works demonstrated that participants tend to respond to objects’ affordances, and a variety of behavioral, brain imaging, and TMS studies with painful and dangerous stimuli were carried out in the domain of pain investigation. These two lines of research were merged in some recent behavioral studies on affordances and dangerous objects. Interestingly, recent evidence revealed that not only pleasant and neutral objects but also dangerous objects activate motor information during our interaction with them. In previous investigations (Anelli et al., 2012a,b), we studied resonance mechanisms activated during the observation of somebody in potential interaction with a dangerous object. We used a priming paradigm with both school-age children and adults, so that participants observed a hand or a control object followed by a neutral or dangerous object. Results revealed that, irrespective of age, motor responses were slower with neutral objects than with dangerous ones, indicating the emergence of a facilitation effect (affordance effect) with neutral objects and of an interference effect with dangerous objects, probably due to aversive affordances. In addition, in both children and adults, motor resonance mechanisms were activated during the observation of biological hands with respect to non-biological ones. To note, the higher the motor resonance induced by biological hands, the stronger the inhibition registered with dangerous objects. To sum up, these studies can be considered as a proof of the influence of objects dangerousness on the motor responses, when objects were preceded by a hand suggesting a potential interaction with them.

Along this line of research, in a subsequent study (Anelli et al., 2013) we adopted a cued bisection paradigm, in which the line to bisect was flanked by images of objects belonging to different categories (dangerous vs. neutral objects). This allowed us to investigate the influence of objects dangerousness on motor responses with a novel paradigm. We measured in both children and adults whether the performance was biased toward a specific object category, independently from the observation of others’ actions, as happened in our previous studies (Anelli et al., 2012a,b). Results not only demonstrated that participants were sensitive to objects dangerousness, but also that this sensitivity was maintained across lifespan, since both in children and adults the line midpoint was shifted toward the neutral object or, in other words, on the side opposite to the dangerous object. This suggested the existence of two specific effects, namely an affordance effect occurring with neutral objects and an interference/inhibitory effect taking place with dangerous objects. This last effect seems to induce the tendency to “escape” from the dangerous object and to approach the neutral object, which is responsible of the motor response’s bias.

To sum up, recent evidence suggests that even dangerous objects can modulate the motor system evoking aversive affordances, i.e., inducing the tendency to avoid dangerous objects. The research area about aversive affordances represents a new and intriguing research field, since so far the majority of studies with painful and dangerous stimuli strictly concerned the domain of pain investigation.

In the present work we focus on some unanswered questions on aversive affordances, by investigating object dangerousness without considering motor resonance mechanisms, and by exploring whether and how the observation of a neutral or dangerous object, in a static or dynamic situation, can differently modulate our motor responses.

First of all, previous studies investigated how motor responses were influenced by object dangerousness, but limited their focus to situations in which objects were preceded by hands in potential interaction with them, thus generating a motor resonance effect (e.g., Anelli et al., 2012a,b). Instead, in the present work we focused on neutral and dangerous objects processing when no agent was shown, investigating the perception of objects dangerousness independently from the observation of others’ actions and thus from the emergence of motor resonance effects.

Second, in the literature static images are usually presented. Since objects/entities are typically threatening when they approach us, we chose to employ a more dynamic and ecologically rich experimental setting, by showing stimuli in dynamic scenes. This more natural embedding allows us to take into account the spatial relationship between stimuli and subject, and thus to consider dangerousness no longer as an objective property, but as a relational one.

Third, so far some aforementioned studies (e.g., Costantini et al., 2010, 2011a,b) investigated the relation between object features and space by manipulating 3D objects presentation in peripersonal vs. extrapersonal space. In the current study we consider the manipulation of the object size to give a cue indicating distance: when the object’s size is larger this means it is closer to the participant’s body, when it is smaller it means it is further away from the participant.

Fourth, it can be posited that different response modalities subtend different motor actions, and specifically key-releases can underlie withdrawal movements and key-presses can underlie approach movements. So far Morrison et al. (2007b) considered this kind of link between response modalities and motor actions in relation to empathy for pain. Conversely, we are interested in the link between response modalities and objects dangerousness aside from pain. In addition, we intend to explore the effects of different response modalities in a dynamic space, considering whether the tendency to press or to release a key is higher when objects come toward us or when they move away from us.

Finally, even if a couple of studies (Lloyd et al., 2006; Coello et al., 2012; see General Discussion) investigated the relationship between dangerous objects and bodily space, to our knowledge there is no evidence on how information on dangerousness emerges in time. The paradigm we chose allowed us to investigate the time-course of the emergence of the aversive affordance effect, evaluating the necessary time to process dangerousness and to respond to dangerous objects. Indeed, it is possible that we immediately respond to this kind of affordance, as soon as an object appears, or alternatively that we need time to process it and to prepare our motor response, for example to prepare ourselves to escape.

To explore these issues, we conducted three experiments requiring participants to perform a simple categorization task, i.e. to decide whether the stimulus shown was an artifact or a natural object. To respond they were required to either press or release one of two designed keys, observing objects in dynamic (Experiment 1) or static (Experiments 2 and 3) conditions. As in our previous works, we focused on how dangerous and neutral objects are perceived and processed at a motor level. We were not interested in the distinction between risk for pain and threat, but in the motor responses evoked by the observation of objects or entities that can potentially provoke pain, independently of their being active or passive.

The aims of the study and our predictions are the following. First, we aim to investigate the sensitivity to objects dangerousness and the emergence of related affordances without showing somebody in *interaction* or potential interaction with the object, and thus without considering motor resonance mechanisms. We hypothesize a facilitation effect of the motor responses with neutral objects, and an interference effect slowing down responses to dangerous objects, in line with our previous results (Anelli et al., 2012a,b).

Second, we focus on the impact of neutral and dangerous objects when they come toward us (dynamic presentation) or when they are close to us (static presentation) with respect to when they go away from us (dynamic presentation) or when they are distant from us (static presentation).

Third, and related to previous point, we intend to clarify how static and dynamic objects’ presentations influence the response to neutral vs. dangerous objects, and whether there is a modulation due to the response modality (key press vs. release). We hypothesize that motor responses would be facilitated, and thus response times would be faster, with dynamic than with static presentations with dangerous objects and release response, due to the fact that humans might tend to escape from dangerous objects and entities as soon as possible, particularly when they have an aggressive behavior.

Fourth, we investigate the time-course of the process, and thus whether the processing of dangerous objects allows immediate responses or whether it requires to prepare responses, also considering the object’s distance. Indeed, notice that distance and time are related: when we can see dangerous entities from far away, we have time to prepare our responses; this is not the case when these entities are very close to us. We do not advance a precise prediction on this point, but our aim is to examine the time-course of dangerous objects processing.

Finally, in light of our previous studies (Anelli et al., 2012a,b), that focused on the processing of objects typology and objects category both in children and in adults, we decided to explore age-related effects.

## Experiment 1

The aim of the first experiment was to investigate whether participants were sensitive to differences in the direction of object movement. In particular, we intended to verify if observing a dangerous or a neutral object in a dynamic situation (i.e., a video of an object moving away or near to the participant) can differently influence the motor responses. In addition, we considered how motor responses can be modulated by the considered variables at different ages, by testing both children and adults.

To these aims, we ran an experiment in which participants were required to distinguish between an artifact and a natural object, so that the object dangerousness and movement direction were not relevant to the task.

### Method

#### Participants

Fourteen undergraduate students from the University of Bologna (six males and eight females, mean age: 20.7 years, range: 19–27) and 14 children (seven males and seven females; mean age: 11.2 years, range: 10–12), took part in the experiment. All participants were right-handed and had normal or corrected-to-normal vision. All were naive as to the purpose of the experiment and they or their parents, as for children, gave informed consent. The present and the following experiments were approved by the Psychology Department’s ethical committee of the University of Bologna.

#### Apparatus and stimuli

Participants sat in front of a 17″ color monitor (the eye-to-screen distance was approximately 50 cm). E-Prime 2.0 software was used for presenting stimuli and collecting responses.

The experimental stimuli consisted of 16 color pictures of common graspable objects (see Table 1). All objects were large and would normally be grasped with a power grip. There were four categories (dangerous-natural objects, dangerous-artifact objects, neutral-natural objects, neutral-artifact objects), with four objects for each class. The set of objects stimuli was the same used in other studies (Anelli et al., 2012a,b, 2013) in which we asked an independent group of 43 participants to rate on a five-points Likert scale the dangerousness of the target objects. The ANOVA with the factors *Object Typology* (neutral and dangerous) and *Object Category* (artifact and natural) manipulated within-items revealed that there was a significant difference between neutral and dangerous objects [main effect of *Object Typology*, *F* (1, 12) = 95.3, MSE = 0.24, *p* < 0.001].

**Table 1:** **The 16 experimental stimuli**.

	Neutral objects	Dangerous objects
Natural objects	Cat	Porcupine
	Chick	Scorpio
	Plant	Cactus
	Tomato	Husk
Artifact objects	Bulb	Broken bulb
	Glass	Broken glass
	Lighted out match	Lighted match
	Spoon	Knife

#### Procedure

Participants were required to decide whether the stimulus was an artifact or a natural object, so that the *Object Dangerousness* (i.e., dangerous vs. neutral) was totally irrelevant to the task. As soon as the go-signal appeared (i.e., a green circle on the middle part of the object), participants had to respond by using one of two designed keys. Since we manipulated the *Manual Response Typology*, we divided the participants into two groups: the first group had to press the response-key, whereas the second had to release the response-key. Moreover, in both groups half of the participants were required to make a right-hand response if the target was an artifact object and a left-hand response if it was a natural one, whereas the opposite hand-to-category arrangement was applied to the other half.

The experiment consisted of one practice block of 16 trials and one experimental block of 128 trials. Each trial began with a fixation point (+) displayed for 500 ms in the center of the screen. Then, the video of a moving object was shown for 1000 ms and followed by a static picture of the object (of the same size of the last video frame) containing the go-signal (a green circle) that remained on the center of the screen until a response had been made or 2000 ms had elapsed. Participants received feedback on reaction time (RT) after pressing the right or the wrong key (the RT value or “Error,” respectively). The next trial began after the feedback disappeared.

Each object was presented eight times: during half of the presentation the object moved away from the participant, with a progressive zoom out of the object, while in the other half the object moved toward the participant, with a progressive zoom of the object.

Overall the experiment consisted of 144 trials and lasted about 20 min.

#### Data analysis

Reaction times for incorrect responses and RTs more than two standard deviations from each participant’s overall mean were excluded from the analysis.

The correct RTs were entered into a repeated-measures ANOVA with *Object Movement Direction* (away and near), *Object Dangerousness* (dangerous and neutral), and *Object Category* (artifact and natural) as within-subjects factors, and *Manual Response Typology* (press and release) and *Age Group* (children and adults) as between-subjects factors. Fisher’s LSD *post hoc* tests were also conducted on significant interactions.

### Results

**Figure 1:**
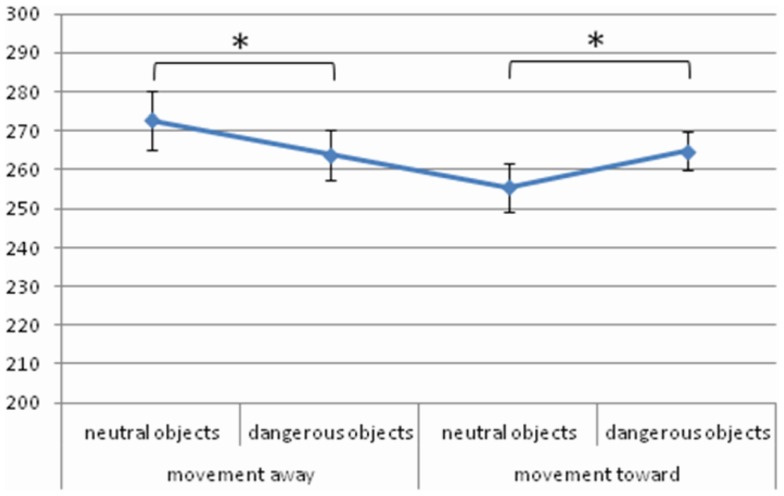
**Significant *Object Dangerousness* × *Object Movement Direction* interaction for RTs in Experiment 1, values are in milliseconds and bars are SEM**.

The interaction between *Object Dangerousness* and *Object Movement Direction* [*F* (1, 24) = 8.14, MSE = 578, Cohen’s *f*  = 0.58, *p* < 0.01, power = 0.78] was significant. *Post hoc* test revealed that when the objects moved toward the participant neutral objects were processed faster than dangerous ones (255 and 265 ms, respectively, *p* < 0.05). In contrast, when the objects moved away from the participant responses to dangerous objects were faster than to neutral ones (264 and 273 ms, respectively, *p* < 0.05) (Figure 1).

**Figure 2:**
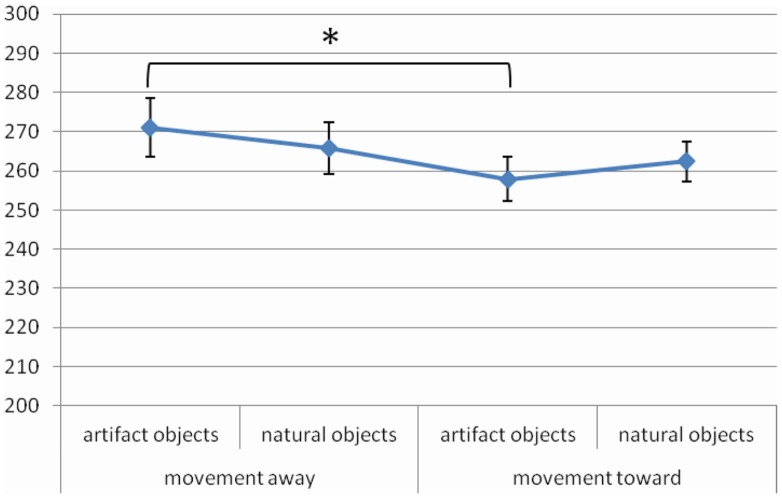
**Significant *Object Category* × *Object Movement Direction* interaction for RTs in Experiment 1, values are in milliseconds and bars are SEM**.

Furthermore, the interaction between *Object Category* and *Object Movement Direction* [*F* (1, 24) = 4.79, MSE = 275, Cohen’s *f*  = 0.45, *p* = 0.04, power = 0.56] was significant. *Post hoc* test revealed that for artifact objects RTs were faster when objects moved toward than away from the participant (258 and 271 ms, respectively, *p* < 0.001) (Figure 2).

There were no other significant main effects or interactions (*p*s > 0.05).

### Discussion

Results showed that, in a dynamic condition, responses were specifically influenced by the object movement direction in a twofold way. First, the movement direction affected the processing of objects belonging to different typologies, since neutral objects were processed faster when moving toward-approaching the participant. This effect can be considered as a dynamic affordance effect. In contrast, we found that dangerous objects were processed faster when moving away from the participant. The longer RTs with dangerous objects when they approached participants are probably due to a blocking effect. We will discuss this issue more thoroughly in the Section “General Discussion.”

Second, the object movement direction also modulated the processing of object category, since responses to artifact objects were faster when moving toward-approaching the participant. This finding adds to previous one as a further demonstration of a dynamic affordance effect, which emerges with a specific category, that of artifact objects. As shown in previous studies, this is likely due to artifact objects activation both of the tendency to manipulate them and to use them, differently from natural objects that convey only information related to manipulation (e.g., Borghi et al., 2007; Vainio et al., 2008; Anelli et al., 2010; Jax and Buxbaum, 2010).

To note, in this experiment we did not register any influence of manual response typology on motor responses. This could either indicate that the employed types of manual response are not effective or that the absence of effects can be rather attributed to the modality of stimuli presentation. We favored the latter interpretation that would imply that, in a dynamic condition, the movement direction of objects became more important than the different motor responses (i.e., press vs. release) at the disposal of participant. The next experiments will allow us to verify these two alternative hypotheses, since we presented objects in a static condition. If, in line with our second explanation, in Experiments 2 and/or 3 the effect of manual response typology will be present, this would mean that it effectively has a different role depending on the modality of objects presentation.

A final point deserved our consideration: the lack of influence of different age classes we considered, namely children and adults. These data allowed us to speculate that object dangerousness represents a salient object’s property, probably because it is adaptive to learn to quickly distinguish between neutral and dangerous objects early on during development. This explanation fits well also with previous evidence on school-age children showing their early sensitivity to object dangerousness and the emergence later in life of more subtle differences, such as those related to object category (Anelli et al., 2012a). On the basis of the results of Experiment 1 and on previous evidence, we have good reasons to predict that the factor age will not influence the results. For this reason, even if it is not possible to completely exclude any effects of age, in the following experiments we will not take into account different age classes, but the sample will be constituted only by adults.

## Experiment 2

Experiment 2 was aimed at understanding what happened when participants observed dangerous or neutral objects in a static situation, rather than in a dynamic one. The task was the same of the previous experiment, i.e. participants were required to distinguish between an artifact and a natural object. To note, in order to explore the time-course of dangerousness processing, participants had time to process objects and to prepare their motor responses: we presented a static picture of an object for 1 s before the appearance of another static picture of the same object containing the go-signal to respond. As explained above, here and in the next experiment we collected only data on adults.

### Method

#### Participants

Sixteen undergraduate students from the University of Bologna (3 males and 13 females, mean age: 19.8 years, range: 19–25) took part in Experiment 2 for course credits. All participants were right-handed and had normal or corrected-to-normal vision. All were naive as to the purpose of the experiment and gave informed consent.

#### Apparatus, stimuli, and procedure

The stimuli and the task were the same of previous experiment. However, in the present experiment, participants observed the objects in a static situation, whereas during Experiment 1 the objects were presented in a dynamic situation.

The experiment consisted of one practice block of 12 trials and one experimental block of 192 trials. Each trial began with a fixation point (+) displayed for 500 ms in the center of the screen. Then, the static picture of an object was shown for 1000 ms and followed by another static picture of the same object containing the go-signal (a green circle) that remained on the center of the screen until a response had been made or 2000 ms had elapsed. Participants received feedback on RT after pressing the right or the wrong key (the RT value or “Error,” respectively). The next trial began after the feedback disappeared.

Each object was presented 12 times: in one-third of the trials the object with the go-signal was larger than the first static picture (big size condition), in one-third it remained of the same size of the first static picture (normal size condition), and in the other-third it was smaller than the first static picture (small size condition). In the big size and in the small size conditions, the object with the go-signal had the same size of the last frame of the video clip shown in Experiment 1 (toward and away conditions, respectively).

Overall the experiment consisted of 204 trials and lasted about 25 min.

#### Data analysis

The data were treated according to the same criteria used for Experiment 1. RTs for incorrect responses and RTs more than two standard deviations from each participant’s overall mean were excluded from the analysis.

The correct RTs were entered into a repeated-measures ANOVA with *Object Size* (big, normal, and small), *Object Dangerousness* (dangerous and neutral), and *Object Category* (artifact and natural) as within-subjects factors, and *Manual Response Typology* (press and release) as between-subjects factor. Fisher’s LSD *post hoc* tests were also conducted on significant interactions.

### Results

**Figure 3:**
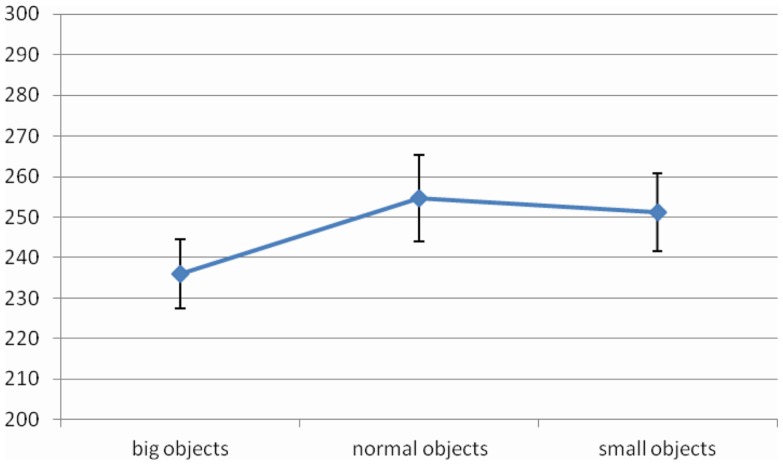
**Significant *Object Size* effect for RTs in Experiment 2, values are in milliseconds and bars are SEM**.

**Figure 4:**
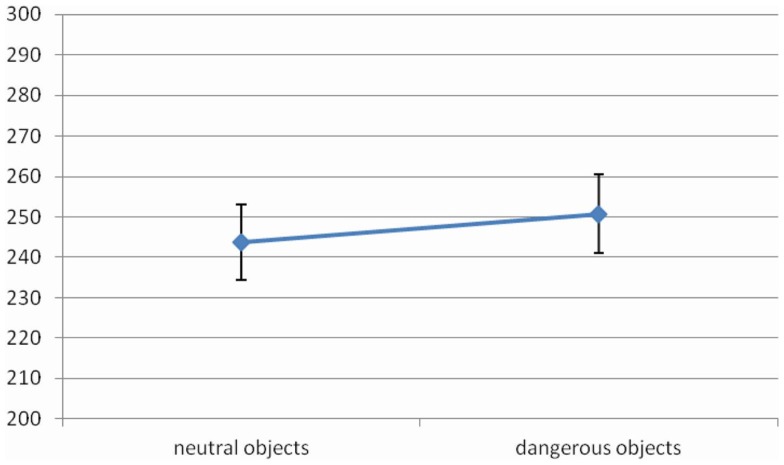
**Significant *Object Dangerousness* effect for RTs in Experiment 2, values are in milliseconds and bars are SEM**.

The main effects of *Object Size* [*F* (2, 28) = 8.05, MSE = 781, Cohen’s *f*  = 0.75, *p* < 0.01, power = 0.93] and *Object Dangerousness* [*F* (1, 14) = 5.70, MSE = 412, Cohen’s *f*  = 0.64, *p* = 0.03, power = 0.60] were significant. RTs were faster when the object was big rather than normal and small (236 vs. 255 vs. 251 ms, respectively) (Figure 3). Moreover, participants were faster when the object was neutral rather than dangerous (244 vs. 251 ms, respectively) (Figure 4).

### Discussion

Results revealed that participants were sensitive to the objects’ dangerousness, as response times were faster with neutral than with dangerous objects. In line with our hypothesis, this evidence pointed out the influence of a fine object property such as object dangerousness on motor responses. In line with our previous data (Anelli et al., 2012a,b), motor responses were facilitated when participants were faced with neutral objects, while they were slowed down with dangerous objects, probably due to an interference effect. It is worth to underline that here we replicated the emergence of an aversive affordance effect independently from the influence of hand’s presentation and thus exclusively by means of the object presentation.

In addition, we registered an effect of the objects’ size, as response times were faster with big than with normal and small objects. Two explanations were possible. The first referred simply to a perceptual effect, so that larger objects were processed faster than smaller ones. The second, and more interesting to us, explained this effect not only as visual but as motor as well. In this latter case, objects would evoke faster motor responses since grasping larger objects is less complex than grasping smaller ones (e.g., Bazzarin et al., 2007; Ranzini et al., 2011). One possible way to disentangle this point was to rely on time: one could hypothesize that this size effect may be affected by the time that a participant has to respond. In fact, in the current experiment participants had sufficient time (1 s) to process different objects’ features and to prepare their motor responses. Conversely, in the next experiment participants will not have such a time interval, but they will have to respond immediately as soon as the object appears. If, in line with our second explanation, in Experiment 3 the effect of size will not be present, this would mean that the effect we found in Experiment 2 was only perceptual/visual. The absence of an interaction with the object dangerousness did not allow us to determine whether this supposed motor effect was linked to the object dangerousness. However, one could hypothesize that, in the case of dangerous objects, some time is needed to prepare ourselves to escape from them. If in Experiment 3 we will register an interaction between objects size and objects dangerousness, this would allow us to determine that a specific motor response for neutral and dangerous objects (i.e., grasping and escaping, respectively) emerges, hence demonstrating that the effect registered in Experiment 2 was not only perceptual but motor as well. This result would be also in line with data of Experiment 1 showing the emergence of a facilitation effect with neutral objects and of an escaping effect with dangerous ones.

One final aspect is worth noticing: we did not find any effect of the object category, in contrast with previous experiment and with the majority of the studies on this issue. Even if we cannot say much about a null result, we can speculate that this was due to the fact that the distinction between dangerous and neutral objects was much more salient, and washes out the distinction between an artifact and a natural object.

## Experiment 3

Experiment 3 was a control experiment. The only difference from Experiment 2 was that participants were required to discriminate between an artifact and a natural object as soon as the object appeared on the screen, so that an immediate coding of the stimulus was required. Along with previous data, this manipulation allowed us to verify the time-course of sensitivity both to objects dangerousness and objects size, and to clarify the motor vs. perceptual features of the effect size emerged in previous experiment.

### Method

#### Participants

Sixteen undergraduate students from the University of Bologna (5 males and 11 females, mean age: 20.3 years, range: 19–26) took part in Experiment 3 for course credits. As in previous experiments, all subjects were right-handed and had normal or corrected-to-normal vision. All were naive as to the purpose of the experiment and gave informed consent.

#### Apparatus, stimuli, and procedure

The apparatus and stimuli were the same used in Experiment 2. The only difference was that participants were instructed to respond as soon as the object appeared.

Each trial began with a fixation point (+) displayed for 500 ms in the center of the screen. Soon after, the static picture of an object containing the go-signal (a green circle) was shown until a response had been made or 2000 ms had elapsed. Participants received feedback on RT after pressing the right or the wrong key (the RT value or “Error,” respectively). The next trial began after the feedback disappeared.

Each object was presented 12 times: in one-third of the trials the object with the go-signal was large (big size condition), in one-third it had a normal size (normal size condition), and in the other-third it was small (small size condition). In the big size and in the small conditions, the object with the go-signal had the same size of the last frame of the video clip showed in Experiment 1 (near and away conditions, respectively) and of the second object showed in Experiment 2.

Overall the experiment consisted of 204 trials and lasted about 25 min.

#### Data analysis

The data were treated according to the same criteria used for previous experiments. RTs for incorrect responses and RTs more than two standard deviations from each participant’s overall mean were excluded from the analysis.

The correct RTs were entered into a repeated-measures ANOVA with *Object Size* (big, normal, and small), *Object Dangerousness* (dangerous and neutral), and *Object Category* (artifact and natural) as within-subjects factors, and *Manual Response Typology* (press and release) as between-subjects factor. Fisher’s LSD *post hoc* tests were also conducted on significant interactions.

### Results

**Figure 5:**
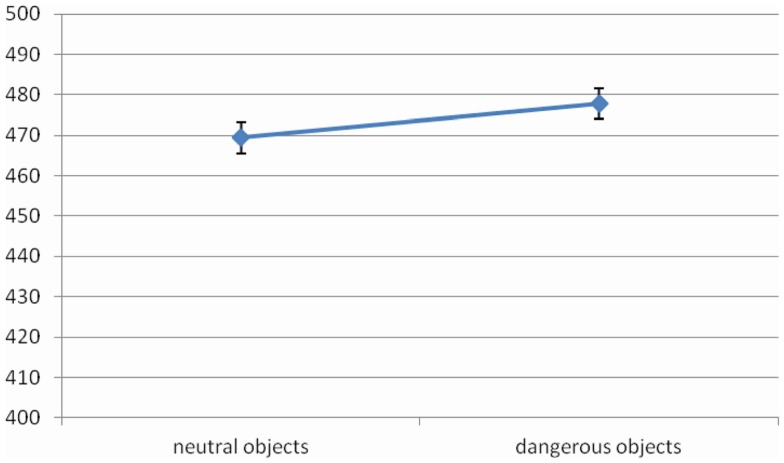
**Significant *Object Dangerousness* effect for RTs in Experiment 3, values are in milliseconds and bars are SEM**.

The main effect of *Object Dangerousness* [*F* (1, 14) = 10.08, MSE = 337, Cohen’s *f*  = 0.85, *p* < 0.01, power = 0.84] was significant. RTs were faster when object was neutral rather than dangerous (469 vs. 478 ms, respectively) (Figure 5).

**Figure 6:**
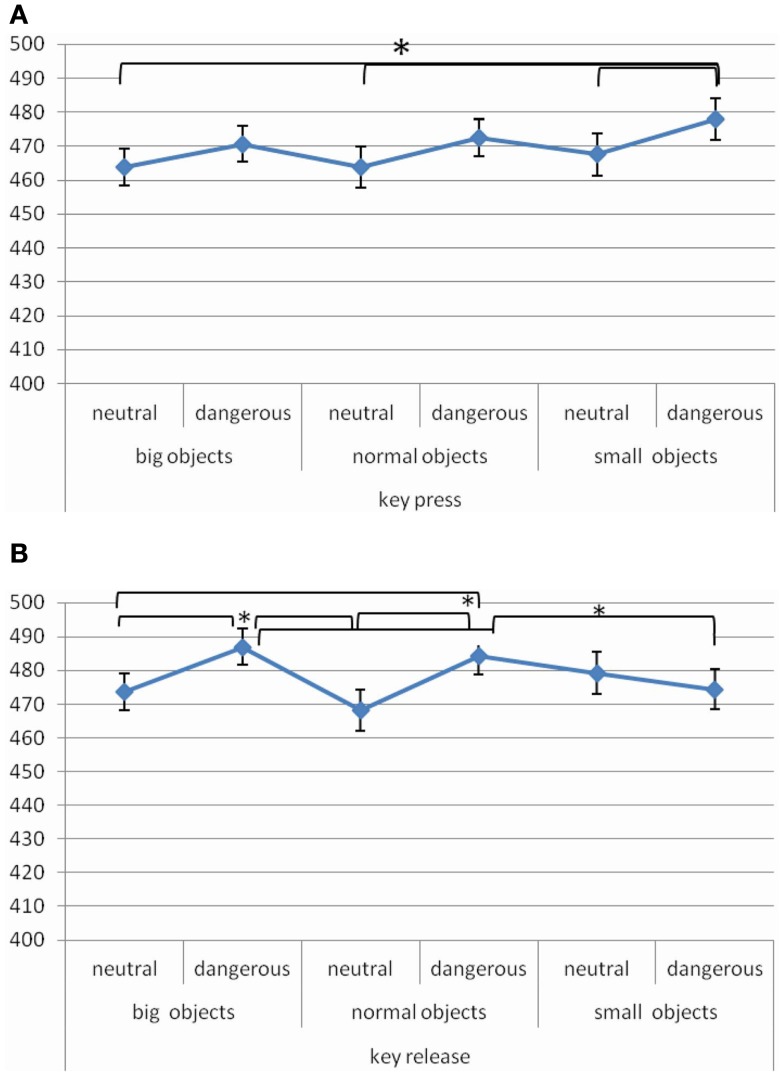
**Significant *Manual Response Typology* × *Object Size* × *Object Dangerousness* interaction for RTs in Experiment 3 [(A) key press manual response typology; (B) key manual release response typology], values are in milliseconds and bars are SEM**.

The interaction between *Manual Response Typology*, *Object Size*, and *Object Dangerousness* [*F* (2, 28) = 3.64, MSE = 182, Cohen’s *f*  = 0.52, *p* < 0.05, power = 0.62] was significant. *Post hoc* test showed that when the task required to press the key, responses were faster when the object was neutral big, neutral normal, and neutral small than dangerous small (464 vs. 478 ms, *p* < 0.01; 464 vs. 478 ms, *p* < 0.01; 468 vs. 478 ms, *p* < 0.05, respectively) (Figure 6A). Moreover, when the task required to release the key, responses were faster in the following comparisons: (i) when the object was neutral big and neutral normal than dangerous big (474 vs. 487 ms, *p* < 0.01; 468 vs. 487 ms, *p* < 0.001, respectively); (ii) when the object was neutral big and neutral normal than dangerous normal (474 vs. 484 ms, *p* < 0.05; 468 vs. 484 ms, *p* < 0.01, respectively); (iii) when the object was dangerous small that dangerous big and dangerous normal (474 vs. 487 ms, *p* = 0.01; 474 vs. 484 ms, *p* < 0.05, respectively) (Figure 6B).

### Discussion

In keeping with what found in Experiment 2, participants responded faster to neutral objects than to dangerous ones.

Interestingly, participants were not sensitive to the objects’ size, in contrast to what happened in Experiment 2. Most crucial for us was the significant interaction between *Manual Response Typology*, *Object Size*, and *Object Dangerousness*. With key press responses small dangerous objects were slower than neutral objects. This would suggest that the facilitation due to the tendency to grasp objects, as revealed by the key press responses associated to approach movements, was stronger with neutral objects, probably due to an affordance effect. In addition, with key release responses small dangerous objects were faster than other dangerous objects. This indicated that the interference due to the tendency to escape from dangerous objects, as revealed by the key release responses associated to withdrawal movements, was particularly marked when dangerous objects were large, thus closer to us. This can be due to the fact that, when the object is still far away from us, we can react moving away from it, while when the object is close to us a blocking effect is present. This blocking effect can be qualified in two different ways, both compatible with our results. First, dangerous stimuli could elicit freezing. The freezing behavior, i.e. the tendency to persist in an immobile state in front of aversive stimuli when there is no way to escape, has been documented in animals such as rats (e.g., Bolles and Collier, 1976). Alternatively, the presence of aversive stimuli could slow down dramatically motion speed inducing to perform more careful and cautious movements. Our data do not allow us to disentangle between these two different strategies, of which the first is probably more instinctive, the second more intentional. For these reasons we will refer more generically to a blocking effect. Importantly, the current experiment clarified that this blocking effect with dangerous objects was not present only with key press but also with key release responses. In sum, the interaction revealed the presence both of an affordance effect and of an aversive affordance effect, and was therefore in line with results of Experiment 1. Importantly, these results also demonstrated that the type of stimuli presentation (dynamic vs. static) influenced the manual response typology. These points will be discussed in the next section.

In addition, this interaction helped to interpret the results of the previous experiment clarifying that the size effect was not simply a perceptual one, but was motor as well. Indeed, it seemed to imply the tendency to grasp neutral objects and to escape from dangerous objects.

Further, the difference between Experiments 2 and 3 allowed us to speculate that the size effect can be influenced by the time that participants had at their disposal to respond. In fact, when they had a brief delay (1 s) before responding, as in Experiment 2, and thus they can prepare a motor response, it is possible that they process information related to dangerousness and size in a separate fashion. On the contrary, when an immediate response was required, and thus participants cannot prepare a motor response, as in Experiment 3, they could process information on dangerousness in strict relation to information on size. This indicated that participants were able to integrate different kinds of information rather quickly when rapid responses were required.

## General Discussion

In the present study we investigate whether motor responses are influenced by the observation of object dangerousness in dynamic and static situations, without showing a direct or potential interaction between an object and an effector. In three experiments we focused on the conceptual distinction between neutral and dangerous objects, by asking participants to perform a simple categorization task (i.e., to decide whether the stimulus shown was an artifact or a natural object), by pressing or releasing one of two designed keys. The object size was manipulated in order to provide a cue indicating distance, namely smaller objects indicated objects more distant from the participant’s body, whereas larger objects indicated objects closer to the participant. The object presentation could be dynamic (Experiment 1) or static (Experiments 2 and 3), as objects moved toward or away from participants (dynamic presentation) or objects were close or distant from participants (static presentation). Moreover, time-course has been considered, by investigating whether the processing of dangerousness differed depending on whether time to prepare motor responses was given (Experiments 1 and 2) or whether an immediate motor response was required (Experiment 3).

In Experiment 1 both children and adults were tested, while in Experiment 2 and 3 the sample was composed only by adults. Despite the complexity of our experimental design, our results are quite consistent across experiments. We will discuss them below.

First of all, results of all three experiments showed that participants were sensitive to the difference between dangerous and neutral objects, in line with our previous data (Anelli et al., 2012a,b). In particular, dangerous objects produced an interference effect, whereas neutral objects produced a facilitation effect, as we registered faster RTs with neutral and slower RTs with dangerous objects. Neither of the two effects was modulated by the manual response typology (key press vs. release). Interestingly, a recent study (Witt and Sugovic, 2013) demonstrated that threatening objects seemed to move faster than non-threatening ones, and that objects easier to block appeared to move slower than objects more difficult to block.

The present work allowed us to advance some speculations about the possible neural mechanisms involved in the processing of neutral and dangerous objects. To note, differently from previous behavioral and TMS studies (e.g., Avenanti et al., 2005; Morrison et al., 2007b; Anelli et al., 2012a,b), in the present study we did not present objects in real or possible interaction with a hand. Even if we cannot completely exclude that observing an object could induce the imagination of a hand interacting with it, we are certain that our stimuli did not directly induce resonance mechanisms, since no hand interacting with objects was presented. This allowed us to ascribe the interference and facilitation effects only to the objects’ processing, whose underlying neural basis is represented by the canonical neuron system, and not to the emergence of resonance mechanisms due to the activation of the mirror neuron system. Indeed, researches on object observation (where only objects were shown), as the present one, highlighted the probable involvement of the canonical neuron system (i.e., neurons activated during both the execution of specific object-directed actions and the mere visual observation of the same objects; for a review, see Rizzolatti and Craighero, 2004). To date, it remains unclear whether the canonical system is not only responsible of the affordance effect, but also of the avoidance effect emerged with dangerous objects.

The second interesting result of our study concerns the influence of the spatial relationship between stimuli and subject on the objects’ processing. Our data revealed that participants’ responses were influenced by the kind of object movement direction in a dynamic condition (Experiment 1). In particular, neutral objects were processed faster when they moved toward-approached the participant than when they moved away from her. This result seemed to be in keeping with data showing the emergence of affordances only when objects were located within the perceiver’s or observer’s peripersonal space (Costantini et al., 2010, 2011a). Our finding revealed that a dynamic affordance effect may emerge when a neutral object moved toward/approached the participant. In fact, when an object is at our disposal we can easily simulate to interact with it, provided that it can be for example manipulated or used without any problem, exactly as in the case of neutral objects. In addition, in the dynamic condition we found that a different motor response emerged with dangerous objects, probably due to the activation of an escaping/avoidance mechanism when they move away from the participant.

On the whole, these findings demonstrated the emergence of selective motor effects related to different objects typologies, modulated by the object movement direction in the space and not by actions performed by an observed agent. It is worth noting that in this way dangerousness was considered as a relational property of objects, namely as a property which is neither of the object/environment nor of the acting organism, in keeping with the definition of affordances as intrinsically relational properties.

These results can be of particular interest since so far, to our knowledge, even if a number of studies investigated the relationship between object affordances and space (e.g., Costantini et al., 2010, 2011a,b), there was only sparse evidence on the relationship between dangerousness and space. In a recent study Coello et al. (2012) focused on the perception of reachable space and demonstrated that the size of such space was influenced by the specific level of objects’ dangerousness: they found a significant reduction of the peripersonal space when the threatening part of dangerous objects was oriented toward the participants, with respect to when it was oriented away from them. The impact of the interaction between body and objects on the boundary of peripersonal space suggests an involvement of processes responsible for the simulation of the consequences that some kind of action upon objects can have for us. Notice that in the study by Coello et al. (2012) the stimuli presentation was not dynamical. As to the neural basis of the relationship between dangerous objects and space, a fMRI study of Lloyd et al. (2006) investigated how aversive objects were processed in peripersonal space. Data showed a significant increase in the activation of posterior parietal area when participants viewed a painful stimulation, with respect to an innocuous one, of a rubber hand in participants’ peripersonal space. This suggested an involvement of this cortex in nocifensive responses to aversive stimuli.

A third result concerned the influence of manual response typology. When the object presentation was dynamic (Experiment 1) motor responses were not influenced at all by manual response typology, raising the possibility that the manipulation employed was not effective. Instead, data of Experiment 3, when the objects presentation was static, demonstrated that this was not the case, since two different effects have been registered. On one hand, a facilitation effect emerged when key press responses concerned neutral objects, probably linked to an affordance effect evoked by this kind of objects with a response modality associated to approach movements. On the other hand, key release responses led to a higher interference effect with large dangerous objects, probably due to a tendency to escape evoked by this kind of objects with a response modality associated to withdrawal movements. This finding was in line with the results of Morrison et al. (2007b) that revealed a specific influence of pain observation on overt motor responses. More specifically, when participants observed a painful stimulation, withdrawal movements were speeded whereas approach movements were slowed down. These results were interpreted as due to a facilitation of the kind of motor responses more suitable for avoiding or withdrawing from the object.

On the whole, findings on manual response typology point out the influence of the modality of objects presentation on the emergence of facilitation and interference effects. In fact, in the dynamic condition the movement direction of objects becomes more salient for the participants than their own actions. Conversely, in the static condition the manual response typology becomes relevant, as participants perceived the importance of their own specific actions in order to interact with objects or to avoid them.

As final point, the investigation of the time-course revealed that the processing of dangerousness was influenced by the amount of time that participants had at their disposal to respond. In this respect, the interaction between the three main factors found in Experiment 3, in which no time for action preparation was given, was particularly informative. Indeed, this interaction showed that, while with key press responses small dangerous objects were the slowest items to be processed, with key release responses the slowest items were large dangerous objects. This qualified the interference effect found in previous studies (Anelli et al., 2012a,b) anchoring it to a precise time-course. In fact, when there was no time for action preparation (Experiment 3), the interference effect with dangerous objects was particularly strong with large objects, i.e., when the objects were perceived as near. When the dangerous object was close to us and there was no time for action preparation, a sort of blocking effect occurred.

The situation was quite different when there was time for response preparation (1 s delay). Indeed, in Experiment 2, while the advantage of neutral over dangerous objects was present, there was no interaction and overall large objects were processed faster than small ones. One possibility was that the effect was due mostly to neutral objects; a qualitative analysis suggested this was the case, but the result was far from significance.

The results of Experiment 1 can help us to better comprehend the data. In fact, the advantage of the toward condition (in which the last video frame depicted large objects) was confined to neutral objects, probably due to an affordance effect, i.e. the tendency to grasp neutral objects, that was stronger when objects were approaching. Instead, the advantage of the away condition (in which the last video frame depicted small objects) concerned dangerous objects, probably due to an escaping/avoiding effect. Imagine the following situation: we see from far away a dangerous object/entity, for example a scorpio; given that it is far away, we have some time for action preparation. We immediately start escaping from it. When the scorpio is very close to us, instead, we are afraid, thus we stop and we avoid moving. Combining information on space and time, our results depict a situation similar to the one we have just described.

In the present study we simply presented dangerous and neutral objects, without introducing finer distinctions, for example between threatening and dangerous entities, even if the dynamic presentation suggested a potential threatening effect. Further research is needed to better understand how the motor responses to different kinds of dangerous entities occur in space and time.

## Conflict of Interest Statement

The authors declare that the research was conducted in the absence of any commercial or financial relationships that could be construed as a potential conflict of interest.
